# Incorporation of Cactus Berry (*Myrtillocactus geometrizans*) and Red Prickly Pear (*Opuntia ficus-indica* L. Mill.) Mixtures on Sausages Elaborated from White-Striped Broiler Breast as Possible Natural Antioxidants

**DOI:** 10.3390/foods14244179

**Published:** 2025-12-05

**Authors:** Luis Humberto López-Hernández, Ariadna Abigail Navarro-Olivera, Mariel Calderón-Oliver, Luz Hermila Villalobos-Delgado

**Affiliations:** 1 Laboratorio de Calidad de Carne, CENID Fisiología y Mejoramiento Animal, Instituto Nacional de Investigaciones Forestales, Agrícolas y Pecuarias (INIFAP), km 1.0 carretera a Colón, Ajuchitlán Colón 76280, Querétaro, Mexico; lopez.lhumberto@inifap.gob.mx; 2 Instituto de Agroindustrias, Universidad Tecnológica de la Mixteca, Av. Dr. Modesto Seara Vázquez No. 1, Acatlima, Heroica Ciudad de Huajuapan de León 69004, Oaxaca, Mexico; navariadna@gmail.com; 3 Tecnologico de Monterrey, Escuela de Ingeniería y Ciencias, Av. Eugenio Garza Sada 2501, Campus Toluca, Monterrey 64849, Nuevo León, Mexico; mariel.calderon.oliver@tec.mx

**Keywords:** cactus berry, red prickly pear, sausages, white striping, lipid oxidation, instrumental colour

## Abstract

This study evaluated the potential of cactus berry (C) (*Myrtillocactus geometrizans*) and red prickly pear (P) (*Opuntia ficus-indica* L. Mill.) powder mixtures as natural colourants and antioxidants in chicken batters and sausages made with White Striping (WS) breast meat. The colour and antioxidant activity (AA) of the individual powders and their mixtures–CP (75%C + 25%P), PC (75%P + 25%C), and MCP (50%C + 50%P) were characterised. The mixtures were then incorporated into batters and sausages and compared with controls with and without nitrites. Aqueous extracts of C and P showed the highest total polyphenol and flavonoid contents, showing 7- to 8.5-fold increases over ethanolic extracts. Among the mixtures, PC exhibited the greatest AA, approximately twice that of the others in DPPH^•^, ABTS^•+^, and FRAP assays. In raw batters and cooked sausages, the mixtures enhanced AA and redness, with PC showing the strongest effects. Additionally, MCP maintained the most stable AA and colour for 28 days (1.5 °C). All mixtures also reduced lipid oxidation (TBARS < 1.75 mg MDA/kg) and prevented colour deterioration, achieving performance comparable to nitrites. Overall, C and P mixtures act as effective natural antioxidants and colour enhancers, offering an alternative to synthetic additives for improving the oxidative stability of WS-affected poultry sausages.

## 1. Introduction

The global demand for ready-to-eat foods, particularly sausages, continues to rise. In 2024, sausage production reached 49 million tonnes, of which 16% was derived from chicken meat. Chicken production itself totalled 103.046 million tonnes [1], driven by genetic improvements aimed at achieving faster growth and greater body weight through high-energy diets [2,3]. However, these advances have also led to structural and metabolic abnormalities in muscle broilers, often involving myodegeneration and regeneration [3,4]. As a result, the incidence of breast myopathies has increased, particularly white striping (WS), with prevalence reported as high as 90% [3,5].

WS is macroscopically characterised by white striations parallel to the fibres of the Pectoralis major and thigh muscles. Although not a health risk, it is considered both an aesthetic and technological defect that reduces the value of broiler breast fillets [4,6]. In this sense, fillets showing severe WS may be downgraded in commercial plants and used for manufacturing further processed products (e.g., sausages and nuggets). In contrast, moderate WS fillets are usually marketed for fresh retailing [7]. Although its inclusion in processed products eliminates the problem of aesthetic defects, some studies have shown that WS meat reduces the technological properties of meat and has poor texture [4,7]. To date, only the inclusion of wooden breast meat (another abnormality) has been incorporated in different proportions into sausage and nuggets, showing an increased shear force and binding strength [8,9]. However, the technological properties of WS meat in sausage production remain poorly understood [3,6].

Furthermore, sausages, composed of minced meat, fat, spices, and additives, are exposed to processing and storage conditions that promote oxidative deterioration, compromising quality, shelf-life, and acceptability [10,11]. To prevent or delay meat oxidation reactions, the industry adds synthetic antioxidants such as sodium nitrite, which can inhibit the growth of microorganisms, delay the onset of rancidity, produce cured meat flavour or smell, and stabilise the meat’s red colouration [11,12]. However, this additive has been shown to have adverse effects on consumer health (carcinogenic effect), thereby increasing the demand for natural antioxidants [11,13,14,15,16].

Prickly pears (*Opuntia ficus-indica* L. Mill.), members of the Cactaceae family, thrive in arid regions worldwide. In Mexico, their wide genetic diversity results in cultivars of various colours, including red, violet, green, and yellow [17]. The red prickly pear is consumed fresh and used in beverages [18]. Likewise, the cactus berry (*Myrtillocactus geometrizans*), known locally as bilberry cactus, whortleberry cactus, blue candle, or “garambullo,” produces small red–purple fruits valued for their nutritional and health benefits [19,20,21,22]. Mexico is the leading producer of both fruits, accounting for about 44–45% of global prickly pear production, although official data for cactus berry are not yet available.

These fruits contain high levels of bioactive compounds, especially polyphenols such as betalains, phenolic acids, flavonoids, and anthocyanins [14,17,19,21,23,24]. Red prickly pear extracts have demonstrated technological benefits in meat products by improving colour stability without affecting sensory quality. Furthermore, encapsulated extracts further enhance antioxidant activity and oxidative stability, while pigments contribute to improved colour in raw beef [14,25,26,27].

Despite their promising phytochemical profiles, the use of cactus berry as a functional additive in meat products has not yet been investigated, highlighting a notable gap in the current literature. Therefore, this study evaluated powder mixtures of cactus berry (C) and red prickly pear (P) to assess their effects on pH, lipid oxidation, antioxidant activity and instrumental colour in sausages formulated with WS broiler breast meat during storage.

## 2. Materials and Methods

### 2.1. Chemical Reagents and Food Additives

Trolox ((±)-6-Hydroxy-2,5,7,8-tetramethylchromane-2-carboxylic acid, Schaffhausen, Switzerland), TEP (1,1,3,3-Tetraethoxypropane, Wuxi City, China), Gallic acid (Wuxi City, China), Quercetin (Wuxi City, China), DPPH^●^ (2,2-Diphenyl-1-picrylhydrazyl, Steinheim, Germany), TPTZ (2,4,6-Tris(2-pyridyl)-s-triazine, Schaffhausen, Switzerland), ABTS^•+^ (2,2′-Azino-bis(3-ethylbenzothiazoline-6-sulfonic acid) diammonium salt, Ontario, Canada), and the Folin–Ciocalteu’s phenol reagent (Schaffhausen, Switzerland) were obtained from Sigma Aldrich. Other reagents and solvents were acquired from J.T. Baker (Xalostoc, Mexico). The food additives used were from Fabpsa (Mexico City, Mexico).

### 2.2. Obtaining and Characterisation of Cactus Fruit Products and Their Powder Mixtures

The whole fruits of cactus berry (C) (*Myrtillocactus geometrizans*), including the peduncle, were acquired in the community of Tomilán, Colón, Querétaro, Mexico, in May and June of 2024. The fruits were acquired based on typical eating maturity when the peel is a deep purple colour, and the flesh is slightly firm [28]. Any berry that showed evidence of decomposition or physical deterioration was discarded. The fruits were subsequently washed with drinking water, disinfected with 0.35% colloidal silver in gelatine (Microdyn™, Mexico City, Mexico), and diluted with potable water (1:300, *v*/*v*). Red prickly pears (P) (*Opuntia ficus-indica* L. Mill) were purchased in a market in the city of Querétaro. The fruit peel and the seeds were removed manually in order to obtain the pulp. Fruits (C and P) were ripe enough for consumption. Both C fruits and P pulp were dried separately in a convection oven (ARSA AR-130D, Jalisco, Mexico) for 24 h at 72 °C until reaching a moisture content of 15%. All fruits were ground in a food processor (Newell Brands, FL, USA) and sieved in a 420 μm mesh (W.S. Tyler Company, Cleveland, OH, USA). They were then vacuum-packed and stored in a lightproof drawer at room temperature (22 ± 2 °C, 60 ± 3.1% RH) until use. Each fruit was purchased from three different retailers, and each fruit purchase was considered a batch. From these powders, three mixtures were prepared varying the proportion of C fruits and P pulp: 75% C + 25% P (CP); 75% P + 25% C (PC), and 50% C and 50% P (MCP). A summary of details from this section is shown in Table 1.

In order to evaluate the antioxidant properties and colour of the cactus fruits and their mixtures, extracts were prepared using two solvents (ethanol and water). The C and P powder fruits and their mixtures (CP, PC, and MCP) were dissolved in an aqueous medium (distilled water) and ethanol (ethanol, 96%) at a concentration of 0.2 g/mL, stirred for 15 min at room temperature, stirring frequently under darkness conditions. Afterwards, the mixture was gravity filtered on Whatman^®^ No. 1 filter paper to obtain the extracts, which were prepared in triplicate for each batch.

#### 2.2.1. Polyphenols (TPC) and Flavonoid (TFC) Content

The total polyphenol content (TPC) was determined using the Folin–Ciocalteu’s method with slight modifications [29]. A quantity of 1.5 mL of distilled water and 125 μL of the Folin–Ciocalteu’s reagent (2 M) were added to 50 microliters of the extract’s fruits or their mixtures. After 5 min, 375 μL of sodium carbonate (20% *w*/*v*) and 475 μL of distilled water were added. The mixture was agitated and incubated for 40 min at 37 °C. Absorbance was determined at 765 nm with a spectrophotometer (Genesys 10S UV-Vis, Thermo Fisher Scientific, WI, USA). An equivalent standard curve of gallic acid (GAE) was used, and the results were expressed as g GAE/100 g DW sample. The total flavonoid (TFC) content was determined with 500 μL of extract, 2 mL of distilled water, 150 μL of NaNO_2_ (5% *w*/*v*), and 150 μL of AlCl_3_ (5% *w*/*v*). After 1 min of resting, 1 mL of NaOH (1 M) and 1.2 mL of distilled water were added, and the absorbance was measured at 330 nm. All samples were analysed in triplicate. A standard quercetin equivalent (QE) curve was used, and results were expressed as g QE/100 g DW sample [30].

#### 2.2.2. Antioxidant Activity (AA)

Antioxidant activity (AA) was evaluated using the DPPH^●^, ABTS^●+^, and FRAP techniques with some modifications [31]. Regarding the DPPH^●^ assay (2,2-diphenyl-1-picrylhydrazyl, 0.1% methanol), 900 μL of the DPPH^●^ solution and 100 μL of the extract were mixed. After 15 min at 25 °C, the absorbance was read at 517 nm. The ABTS^●+^ technique was performed with 10 μL of the extract and 990 μL of the ABTS^●+^ solution (7 mM, previously oxidised with potassium persulfate). Absorbance was determined at 734 nm after 7 min. Plasma iron-reducing ability (FRAP) was determined with 30 μL of the extract and 970 μL of the FRAP reagent (1:1, of 2,4,6-Tris(2-pyridyl)-s-triazine and FeCl_3_). Absorbance was determined after 7 min at 593 nm. All samples were analysed in triplicate, and results were expressed in μmol equivalent of Trolox/g sample.

#### 2.2.3. Colour Analysis of Mixtures

The colour determination in each extract (C, P, and their mixtures) was measured with a colourimeter (Konica Minolta CR-410, Illuminant D65, Tokyo, Japan), which was calibrated with a white tile (Y = 93.9, x = 0.3133, y = 0.3195). One hundred millilitres of the sample were placed in a low-density polypropylene bag, providing the required 2 cm depth to measure five different points for each sample. Results were expressed on the CIELab scale as lightness (L*), redness (a*), and yellowness (b*). Samples were analysed six times.

### 2.3. Selection, Conditioning and Quality of Broiler Breast Meat

Broiler normal breasts (Normal) and broiler breasts with white striping defect (WS) were obtained from a Federal Inspection Type (TIF) slaughterhouse. Male Cobb-500 broilers (2.24 ± 0.085 kg live weight) from a single supplier (same farm, feeding, age, and management) were used. The birds were slaughtered following the Official Mexican Regulations [32]. The selection of carcasses exhibiting the WS defect in the breast was performed according to a subjective evaluation based on the thickness of the white striations [5]. Then, three individual batches were conducted using a total of 72 carcasses. The carcasses were selected and classified into the following two types of broiler breast meat (BBM): 36 carcasses with WS defect and 36 with no apparent defect (24 total carcasses per batch; 12 Normal and 12 WS). The carcasses were transported to the Meat Quality Laboratory at the CENID Physiology of INIFAP (National Institute of Forestry, Agricultural and Livestock Research) in Querétaro, where the complete breasts (Pectoralis major and Pectoralis minor muscles) were skinned, deboned, and evaluated fresh. The meat was then cubed (2 × 2 × 2 cm) and frozen at −20 °C until use. A summary of details from this section is shown in Table 1.

#### 2.3.1. Subjective Evaluation of Meat with the White Striping (WS) Defect

The determinations described in this section were performed on breast fillets with and without the white striping (WS) defect. The subjective evaluation of WS severity was conducted considering three categories based on the thickness of the white striations: Normal (NOR)—no visible white lines on the fillet surface; Moderate (MOD)—white lines less than 1 mm thick; and Severe (SEV)—white lines greater than 1 mm thick [5]. Breast samples classified as NOR and SEV were selected for further analyses.

#### 2.3.2. Physicochemical Properties

Chemical composition was determined for each type of meat (N and WS defect) following standard methods [33]: moisture (950.46); ash (920.153); crude protein (928.08); crude fat (991.36). The pH of the samples was measured in a suspension of 10 g of sample in 100 mL of distilled water for 2 min [34]. Measurements were taken using a HI9810452 pH meter (Hanna Instruments Srl, Cluj-Napoca, Romania), previously calibrated with pH 4 and pH 7 buffer solutions. Colour determination was carried out directly on six points of the samples using the same colourimeter that was used previously [35].

The shear force was conducted according to the methodology proposed by AMSA [36]. For this determination, the samples were cooked on a grill (Milan Toast Srl, Monza & Brianza, Italy) at 120 °C to reach an internal temperature of 72 °C. The fillets were then cut into cylinders measuring ≈3 cm long and 1 cm thick, starting from the centre of each muscle (parallel to the muscle fibres). Each cylinder was measured in a TA.XT Plus Texture Analyser equipment, equipped with a Warner-Bratzler “V” cutting device (Stable Micro Systems Ltd., Surrey, UK), with the results reported in kilograms force (kg f). Samples were analysed in triplicate.

#### 2.3.3. Antioxidant Activity

Breast meat extracts were prepared with some modifications. A 2.5 g meat sample was homogenised with 7.5 mL of ethanol for 1 min using an Ultraturrax (digital IKA T18, Staufen, Germany) and centrifuged at 5000 rpm for 15 min (Eppendorf 5804R, Leipzig Germany) to obtain the supernatant [37]. The antioxidant capacity was measured using the 2,2-diphenyl-1-picrylhydrazyl (DPPH^●^) method with some modifications [31]. An aliquot of 100 μL from the extract was taken, to which 900 μL of the DPPH^●^ solution was added. Absorbance was determined at 517 nm by a spectrophotometer (Genesys 10S UV-Vis, Thermo Fisher Scientific, WI, USA) after 15 min of incubation at 25 °C. Samples were analysed in triplicate. The results were expressed in μmol Trolox equivalents per gram of sample. Antioxidant activity was complemented using the FRAP and ABTS^●+^ methods, as previously mentioned in Section 2.2.1.

#### 2.3.4. Lipid Oxidation

The thiobarbituric acid reactive substances (TBARS) method was used to determine lipid oxidation [38]. A 5 g sample was homogenised with 25 mL of distilled water for 2 min. Subsequently, 47.5 mL of distilled water and 2.5 mL of 4 N HCl were added, and the mixture was then combined 1:1 with 0.02 M TBA and heated at 95 °C for 5 min. Absorbance was measured at 538 nm, and TBARS values were expressed as mg malondialdehyde (MDA) per kg of sample. 1,1,3,3-Tetraethoxypropane (TEP) was used to prepare the standard curve. Samples were analysed in triplicate.

### 2.4. Preparation and Evaluation of Batters and Sausages

The meat products were prepared following established methodologies [26,39]. Batters (raw meat emulsion, uncooked) and sausages (cooked batters) were prepared with one of two types of broiler breast meat (BBM): normal breast (Normal) or white striping breast (WS). For this, five treatments were established for the batters, and these batters were subsequently cooked to obtain the sausages. In the preparation of the batters, the different powder mixtures (CP, PC or MCP) were applied at a concentration of 5% (*w*/*w*) of the formulation. Other treatments (CTL) only included the minimal formulation, and the last treatment was the addition of nitrites at 120 mg/kg (NOS). A summary of details from this section is shown in Table 1. The treatments were applied to a minimal formulation (without the use of spices and dyes): 50% chicken meat (Normal or WS), 30% pork lard (1.1 ± 0.08 mg MDA/kg), 17.4% ice, and 2.6% food additives (2% common salt, 0.3% sodium phosphate, and 0.3% carrageenan). The process was carried out in homogeniser equipment (Foss 2094, FOSS A/S, Hilleroed, Denmark) at a low speed for 12 min, with emulsification lasting 3 min. The batters were evaluated immediately. For cooked sausages, each raw batter was stuffed into cellulose casings with a 20 mm diameter (Viscofan de Mexico, San Luis Potosi, Mexico) and formed into 10 cm links. The batters were cooked until they reached an internal temperature of 72 °C, then immediately cooled in an ice bath. The casings were removed before vacuum-packing (1.3 bars, TORREY^®^, Nuevo Leon, Mexico) with six pieces per bag (20 × 15 cm). The packages were then stored at 1.5 ± 0.3 °C in a display with transparent glass doors at 67% RH. Sampling was conducted every 7 days (days 1, 7, 14, 21, and 28) by removing a complete package of sausages.

The batters (BCTL, BCP, BPC, BMCP, and BNOS) were analysed for antioxidant activity, instrumental colour, and lipid oxidation (TBARS). Sausages (SCTL, SCP, SPC, SMPC, and SNOS) were subjected to pH, instrumental colour, and lipid oxidation analysis. Samples were evaluated on days 1, 7, 14, 21, and 28 of the storage period in triplicate.

#### 2.4.1. Physicochemical Characterisation

The pH measurements were carried out as described in Section 2.3.2. The surface colour of batter emulsion and sausages was evaluated using a Konica Minolta CR-410 colourimeter (as described in Section 2.2.3). A 100 g sample of the batter was placed in a polyethene bag with a thickness of 2 cm for the determination. For the sausages, three pieces were finely chopped. Colour measurements were taken at six points per sample, and the results were expressed as L* (Lightness), a* (redness), and b* (yellowness), along with Chroma (C*) [a*^2^ + b*^2^)1/2] and Hue-angle (H°) [tan^−1^ (b*/a*)]. Lipid oxidation by the TBARS method was determined according to Section 2.3.4.

#### 2.4.2. Antioxidant Activity

The batters and sausages were processed to obtain the meat extracts according to Section 2.3.3, and subsequently, the antioxidant activity was evaluated in triplicate.

### 2.5. Statistical Analysis

All samples were analysed in triplicate, except the colour measurement, which was evaluated six times. A one-way analysis of variance (ANOVA) was performed to characterise the aqueous and ethanolic extracts from the fruits (C and P) and their mixtures (CP, PC, and MCP) with regard to TPC, TFC, AA, and instrumental colour. A t-student test was conducted based on the type of meat (Normal and WS) in broiler breast meat to assess physicochemical properties and antioxidant activity. Additionally, the results from analyses of batter emulsions and sausages (AA, instrumental colour, pH, and TBARS) were subjected to a one-way ANOVA to evaluate the effect of the mixtures (CP [BCP, SCP]; PC [BPC, SPC]; MCP [BMCP, SMCP]) and controls (CTL [BCTL, SCTL]; NOS [BNOS, SNOS]). In the sausages, the effect of time (days 1, 7, 14, 21, and 28) among treatments was considered. Significant differences between means were determined using Duncan’s test (*p* ≤ 0.05) in STATISTICA software, version 10.

## 3. Results and Discussion

### 3.1. Total Polyphenol Content (TPC), Total Flavonoid Content (TFC), and Antioxidant Activity (AA) of Cactus Fruit Powders and Their Mixtures

The TPC, TFC, and AA of cactus fruits are presented in Table 2. The aqueous extracts of cactus berry (C) and prickly pear (P) exhibited the highest polyphenol contents (1.4 g GAE/100 g and 1.7 g GAE/100 g, respectively) as well as flavonoid contents (both 0.6 g QE/100 g). Notably, red prickly pear contained significantly more TPC than cactus berry (*p* < 0.05), whereas TFC was similar for both extracts. Prickly pear is rich in phenolic compounds, particularly flavonoid derivatives, with isorhamnetin-based flavonols being the most abundant [17].

Previous studies [13,24,40,41] suggest that fruit composition varies according to the type of phytochemicals present at harvest, which are influenced by factors such as variety, growing conditions, maturity, and extraction process (solvent-to-sample ratio, solvent type, pre-treatment of raw material, interferences, pH, temperature, and extraction time). In the present study, differences in solvent polarity and extraction time may have affected TPC and TFC, potentially explaining the discrepancies with other studies reporting higher or lower values. Flavonoids such as catechins, proanthocyanidins, and condensed tannins are often more efficiently extracted with water [15], which is consistent with the higher phenolic yield obtained from aqueous extracts in this research. Additionally, pre-processing steps such as grinding and drying have been associated with compositional changes that accelerate the loss of phenolic compounds [21]. Cactus berry has been reported to contain up to 13 flavonoids compared with only 6 phenolic acids, indicating its richness in flavonoids such as quercetin, epigallocatechin gallate, and epicatechin [19,22,42]. By contrast, flavonoid levels in prickly pear pulp are lower than those in the peel, with only traces of compounds such as quercetin and its derivatives, rutin, catechin, and flavan-3-ols being detected [17,43,44]. In this study, the TPC and TFC values of prickly pear were comparable to previously reported ranges of 0.022–0.23 g GAE/100 g for TPC and 0.28 g QE/100 g for TFC [43]. For ethanol/water extractions, TPC and TFC were 0.37 g GAE/100 g and 0.15 g QE/100 g, respectively, with dietary fibre content strongly influencing extraction efficiency [14].

The antioxidant activity of cactus fruits is summarised in Table 2. Specifically, the aqueous extract of red prickly pear exhibited the highest values in DPPH^●^ and ABTS^●+^, while cactus berry in ethanolic medium displayed the highest FRAP activity (*p* < 0.05). The antioxidant activity of polyphenolic compounds depends on the redox potential of their hydroxyl groups and on structural interactions among different molecules. Such interactions contribute to antioxidant mechanisms by scavenging free radicals, chelating metal ions, and eliminating oxygen-reactive species [10,45]. Consequently, it is difficult to establish a direct relationship between specific bioactive compounds (e.g., vitamins, carotenoids, betalains, anthocyanins, tocopherols, carbohydrates, among others) and their overall AA [41]. In both fruits, AA is also associated with ascorbic acid and betalains, which are capable of counteracting, reducing, and repairing oxidative stress damage [44,46]. Moreover, the degree of ripeness influences AA: immature fruits show activity mainly due to phenols, flavonoids, and ascorbic acid, whereas overripe fruits present reduced levels of these compounds but retain or even increase betalains [20,24]. Betalains are recognised for their strong antioxidant properties [19,44,47]. In cactus berry, betalains (mainly beta-cyanins) account for about 90% of total antioxidant activity, while phenolic compounds and vitamin C contribute the remaining 10% [20]. Flavonoids such as isorhamnetin glycosides, rutin rhamnosylglucoside, and kaempferol-7-O-neohesperidoside have also been identified in cactus berry, linking its high AA to both ascorbic acid and isorhamnetin glycoside content. Similar flavonoids have been reported in prickly pear (*Opuntia* spp.) fruits and juices [19].

In red prickly pears, betalains and betacyanins have been reported in different concentrations depending on variety [17,23]. Additionally, phenolic acids are present, divided into hydroxybenzoic acids (e.g., cinnamic, chlorogenic, coumaric, ferulic) in higher concentrations, and hydroxycinnamic acids (e.g., 4-hydroxybenzoic, protocatechuic, vanillic, syringic, salicylic, gallic, gentisic) in lower concentrations [19,21,41,44].

For the cactus mixtures in aqueous medium (Table 3), TPC values differed significantly (*p* < 0.05). The PC mixture (aqueous) showed the highest polyphenol content, followed by MCP and CP, highlighting the contribution of prickly pear to TPC. In contrast, the highest flavonoid content (*p* < 0.05) was found in the CP mixture (75% cactus berry/25% prickly pear), while PC and MCP exhibited similar lower values. These results may reflect interactions between phenolic compounds during solubilisation in different solvents, resembling the behaviour observed with hydrophilic solvents [15,48,49].

Regarding the antioxidant activity (AA) in the mixtures, similar trends were observed as in the individual fruits. In aqueous medium, the PC mixture exhibited the highest values in DPPH^●^ and ABTS^●+^ assays, while in ethanolic medium, FRAP activity was higher in the MCP and CP mixtures (*p* < 0.05).

### 3.2. Colour of Cactus Fruit Products and Their Mixtures

The colour parameters of cactus fruits and their mixtures, both in aqueous and ethanolic media, are shown in Table 2 and Table 3, respectively. As expected, instrumental colour measurements (L*, a*, b*) differed significantly (*p* < 0.05) between fruits and mixtures. Since one of the objectives of these mixtures is to impart a reddish hue to white meat sausages, the most favourable results were obtained using water as the extraction medium.

For the individual powders (C and P), luminosity (L*) values were similar between solvents, although they were slightly lower in the aqueous medium (Table 2). Regarding red tones (a*), cactus berry exhibited higher values in an aqueous medium, thus providing a more intense reddish colouration. In contrast, red prickly pear presented the highest yellow tones (b*) in an aqueous medium, while in an ethanolic medium, its values were comparable to those of cactus berry. In the mixtures (Table 3), the a* values increased significantly (*p* < 0.05) in mixtures (aqueous medium) with equal or higher proportions of cactus berry (CP and MCP). The b* values, however, increased in PC due to the contribution of red prickly pear, but decreased in CP and MCP with the inclusion of cactus berry (*p* < 0.05).

These colour changes may be attributed to the possible degradation of phenolic compounds, including betalains, where red prickly pear may contain higher levels of betaxanthins, while cactus berry is richer in betacyanins, which explains the distinct colour tones observed [17,23]. In cactus berry, betalains such as betanin and phyllocactin have been identified at high concentrations and are known to be more polar than anthocyanins. However, ethanol can accelerate betalain degradation because the electron-rich oxygen in the alcohol group promotes nucleophilic attack and the cleavage of conjugated double bonds, particularly under neutral or basic conditions, leading to the hydrolysis of betalains [46].

Post-harvest handling and fruit ripeness are also critical factors influencing pigment content and stability. Betalain levels are affected by water loss after harvest and by their synthesis as a response to oxidative stress [21,46]. Colour parameters in fresh cactus berry fruits, considering ripening stage and agroecological conditions, showed L*, a*, and b* values comparable to those obtained in the present study [20,24]. Additionally, it has been reported that water-soluble red-violet pigments are present at approximately 2.3 mg/100 g in cactus berry and 2.1 mg/100 g in red prickly pear varieties [21]. Based on these findings, cactus berry likely contributed substantially to the pigmentation of the CP mixture.

### 3.3. Broiler Chicken Meat Quality

Table 4 presents the quality characteristics (chemical composition, pH, instrumental colour, and shear force) and antioxidant activity of both Normal and white striping (WS) broiler breast meat. Regarding proximate composition, fat content differed significantly (*p* < 0.05), with WS chicken breasts exhibiting the highest values. Breast myodegeneration in WS is often accompanied by fibrosis and adiposis, which could explain the increased intramuscular fat and the corresponding reduction in protein content [2,4,50]. These results align with findings in WS turkey breasts, where lipid content (Normal 1.04% vs. Severe: 1.38%) increased due to skeletal muscle tissue reorganisation involving connective tissue proliferation and fat infiltration [50]. These changes contribute to the lower relative nutritional value of WS-affected breast muscle.

The remaining proximate parameters were similar between the two types of meat. For normal chicken breasts (Cobb breed), moisture (73.89%) and ash (1.03%) contents were partially consistent with previous findings, although protein (16.95%), fat (6.59%), and pH (6.48) differed [51]. Higher moisture (75.10%) and protein (22.90%) values than those observed in this study have been previously reported [7]. For WS fillets of moderate and severe severity, moisture contents were 75.16% and 74.90%, respectively, while protein contents were 22.20% and 20.90%, respectively.

On the other hand, pH was lower in Normal breasts and higher in WS breasts (*p* < 0.05), consistent with previous findings reporting pH values of 5.80 for normal and 5.90 for WS chicken breasts [52,53]. Elevated pH in WS meat may be associated with enhanced pectoral muscle development and reduced glycolytic potential, which affects post-mortem acidification and results in a higher ultimate pH [2].

In addition, no significant differences were observed in L* between normal and WS breasts. Similar results have been reported, showing unaltered luminosity in WS meat, although extreme pH variations may affect L* [54]. However, slightly lower L* values (56 for normal and 54.9 for WS) have been reported previously [52,53]. Moreover, significant differences (*p* < 0.05) were observed for a*, b*, chroma, and Hue angle: WS breasts had higher a* values and lower b*, chroma, and Hue angle, indicating a redder but less yellow tone. Elevated a* in WS meat may result from severe fibrotic responses, despite potential antemortem stress reducing this parameter [7,54,55]. The higher yellow colouration (b*) in Normal meat could reflect dietary pigment content [54].

Texture analysis revealed significant differences (*p* < 0.05) in shear force, with WS breasts being softer than normal. This contrasts with previous reports indicating that WS meat is less juicy and chewier due to a higher collagen content in heavier fillets [2,4]. In the present study, heat during cooking may have affected collagen-rich connective tissue in WS breasts, reducing shear force values [4,54,56,57].

Additionally, antioxidant activity (AA) was measured by DPPH^•^, ABTS^•+^, and FRAP assays (Table 4). WS meat exhibited the highest AA (*p* < 0.05). Reduced phospholipid content in WS breast fillets [2], which generally promotes lipid oxidation, may influence these results [10]. Additionally, AA, macronutrient content, and bioactive compounds in meat are influenced by diet and genotype [58,59]. Compounds such as α-tocopherol, present in broiler feed, accumulate in muscle and enhance antioxidant potential [60]. Chicken meat also exhibits higher AA than other meats due to histidyl dipeptides (carnosine and anserine) that scavenge free radicals and chelate pro-oxidative metals [31,61]. DPPH^•^ values for normal chicken meat in this study were consistent with previously reported values of 29 μmol Eq./kg meat [31]. The TBARS results are consistent with previously reported results, with severe grade WS meat being more oxidised (0.9 vs. 0.6 mg MDA/kg) than Normal meat, suggesting that the activity of GPx enzyme is low and excessive formation of free radicals [62].

### 3.4. Antioxidant Activity and Colour in Batters

The effect of incorporating cactus berry and red prickly pear mixtures on antioxidant activity and instrumental colour in batters is presented in Table 5.

Within each meat type, most treatments incorporating cactus fruit mixtures significantly increased AA compared with BCTL (*p* < 0.05). For DPPH^•^, the highest responses were observed in both Normal and WS meat with all three mixtures, while for ABTS^•+^, BMCP exhibited the strongest activity in Normal meat. Notably, in the DPPH^•^ assay, Normal meat with mixtures showed similar or higher values than BNOS treatment. Nitrates and nitrites are among the most commonly used curing agents in meat products, with nitrites providing red colouration, a characteristic cured flavour, and exhibiting antioxidant and antimicrobial properties [11,12,63].

In WS meat, the best antioxidant responses were observed with BCP for DPPH^•^ and BPC for ABTS^•+^ compared with BCTL and BNOS. As previously mentioned, cactus fruits contain compounds capable of scavenging free radicals and chelating metals [20,40,46], which may also interact with amino acids and other components in the meat matrix. Furthermore, nitrites contribute to antioxidant activity by chelating metals and participating in redox reactions with meat constituents, forming nitrosyl compounds that exhibit antioxidant properties [11,12].

Regarding instrumental colour, for L*, the base formulation with Normal BBM without colour or antioxidant additives (BCTL) was on average 5 units lower than the results of WS breast. When nitrites were included, L* increased by approximately 2 units, with no differences observed between BCTL and BNOS in WS meat, while changes were noted in Normal batters (*p* < 0.05).

In emulsions with Normal BBM, no differences in L* were observed between BPC and BMCP, although their values were 7–9 units lower than the controls. The CP mixture, with a higher proportion of cactus berry, significantly reduced luminosity in the batters (*p* < 0.05). Red tones (a*) did not differ among the controls (*p* > 0.05). The BCP and BMCP mixtures produced the highest a* values in Normal meat emulsions (*p* < 0.05), whereas in WS meat, a* values were similar across all three mixtures. For the BCTL treatment with WS meat, an unidentified factor during processing or emulsification may have influenced the a* value. For b* values, controls for both meat types exhibited higher yellow tones than batters containing the mixtures (*p* < 0.05). Chroma and Hue angle decreased further with the incorporation of the CP mixture in both meat types. These changes are attributed to the pigments in cactus berry and red prickly pear, primarily betalains, which are mainly responsible for colour [42,44].

### 3.5. Physicochemical Properties of Sausages During Storage

**Figure 1:**
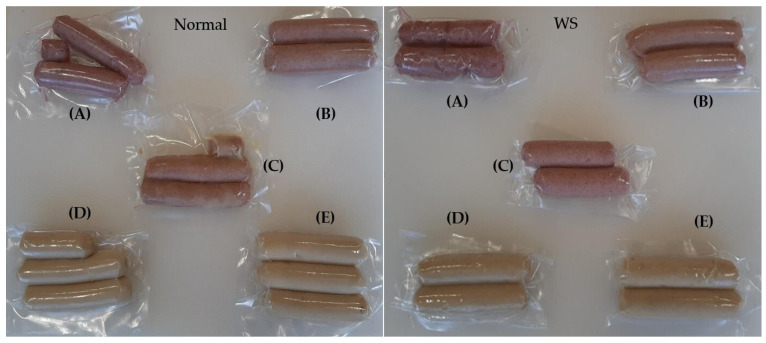
Vacuum-packed sausages made from Normal or White Striping (WS) broiler breast meat: (**A**) SCP (75% cactus berry + 25% red prickly pear); (**B**) SPC (75% red prickly pear + 25% cactus berry); (**C**) SMCP (50% cactus berry + 50% red prickly pear; (**D**) SCTL (control); (**E**) SNOS (nitrites at 120 mg/kg).

Table 6 and Table 7 present the pH and instrumental colour of sausages prepared with Normal and WS broiler breast meat, stored under refrigeration for 28 days. Figure 1 shows sausages made from Normal or White Striping (WS) broiler breast meat.

In sausages prepared with Normal meat, pH in the control treatments (SNOS and SCTL) decreased by day 14 and increased by day 28. However, all cactus mixtures showed an increase in pH by day 28, with no significant differences (*p* > 0.05) among them. A reduction in pH after processing may be influenced by salt content [64]. Although nitrite sources can contribute salt, when accounted for in the overall formulation, nitrites alone do not significantly affect pH [65,66]. Overall, the sausages exhibited higher pH values than the raw meat, consistent with previous findings indicating that cooking can increase pH in meat products [67,68].

Lightness (L*) was initially high and similar in both controls (average 96.0). The cactus mixtures reduced initial L* by 9.7 units (SMCP < SCP < SPC). Despite fluctuations, L* remained relatively stable over time. Redness (a*) in the controls remained low and stable for over 28 days. Among the mixtures, SCP initially showed the highest a* (11.0), decreasing to 8.2 by day 28, remaining higher than SPC (6.8) and SMCP (7.5). Yellowness (b*) decreased in the controls from 18.9 to 12.7 (SCTL) and 8.3 (SNOS), while SCP and SMCP increased from 11.6 to 21.4 and 12.4 to 20.1, respectively. SPC increased slightly from 13.7 to 14.8 and remained stable. Hue angle remained largely unchanged in SCTL, decreased in SNOS, and increased in the mixtures to 69.0 (SCP), 69.5 (SMCP), and 65.3 (SPC) by day 28.

On the other hand, in sausages made with WS meat, pH increased in all treatments by day 28, with SCTL reaching the highest value (7.3), while the other treatments ranged from 6.4 to 6.5. Changes in L* during storage followed a similar pattern to Normal meat, remaining relatively stable throughout the trial. Redness (a*) appeared slightly higher in this meat type (WS), resulting in higher final values after 28 days. For yellowness, although the controls behaved similarly to those with Normal meat, b* did not increase to the same extent in the treatments SCP and SMCP. SCP remained practically constant for b* during storage, whereas SPC increased slightly from 14.3 to 15.9 after 28 days. In this context, the Hue angle was higher in the controls. These results indicate that sausages with cactus mixtures showed redder colour.

### 3.6. Antioxidant Activity of Sausages During Storage

Table 8 shows the antioxidant potential of cactus mixtures in sausages prepared with Normal and White Stripping (WS) broiler breast meat. In sausages made with Normal meat, the SCP treatment induced the highest DPPH^●^ activity on day 1, while antioxidant activity (AA) decreased in all treatments throughout storage. However, SMCP maintained its activity over the 28 days, resulting in higher values than all other treatments (*p* < 0.05).

For ABTS^●+^, although activity increased with the addition of cactus mixtures, SPC and SNOS showed higher values by day 28 (*p* < 0.05), whereas SMCP remained stable during storage. In the FRAP assay, as observed for DPPH^●^, SCP displayed the highest initial activity, which decreased after 28 days of storage; nevertheless, SMCP maintained higher values than the other treatments (*p* < 0.05).

On the other hand, in sausages made with WS meat, the DPPH^•^ assay revealed that the SCP treatment provided the highest antioxidant activity throughout storage, with a similar trend observed for SMCP (*p* < 0.05). Over the same period, the control sausages (SCTL and SNOS) exhibited lower DPPH^•^ activity compared with the cactus mixtures (*p* < 0.05). ABTS^•+^ activity was initially higher in sausages containing cactus mixtures and SNOS; however, after 28 days, SCP and SMCP treatments maintained stable activity levels, showing comparable values to SNOS (*p* < 0.05). During this same period, FRAP values for the SPC treatment were similar to those of SNOS (*p* > 0.05), while the SMCP treatment exhibited the highest AA (*p* < 0.05), which remained stable throughout the 28 days and reached its maximum level at the end of the trial.

### 3.7. Lipid Oxidation (TBARS) of Batters and Sausages

**Figure 2:**
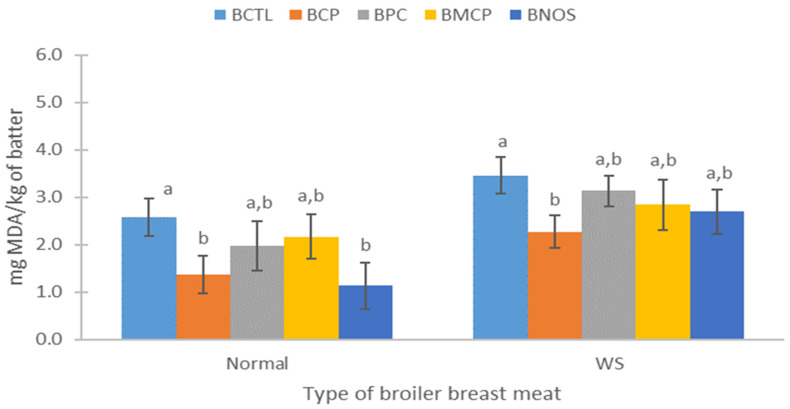
Effect of the incorporation of cactus fruit mixtures (cactus berry and red prickly pear) on lipid oxidation (TBARS, mg MAD/kg meat) of batters made with normal or White Striping (WS) broiler breast meat, n = 9. BCTL: batter made from broiler breast meat, without adding mixtures of cactus fruits (negative control). BCP: batter made from broiler breast meat, with the addition of CP. BPC: batter made from broiler breast meat, with the addition of PC. BMCP: batter made from broiler breast meat with the addition of MPC. BNOS: batter made from breast meat chicken with the addition of 120 mg of nitrites/kg of batter. ^a,b^: Different letters between treatments in the same type of broiler breast meat indicate differences (*p* < 0.05).

**Figure 3:**
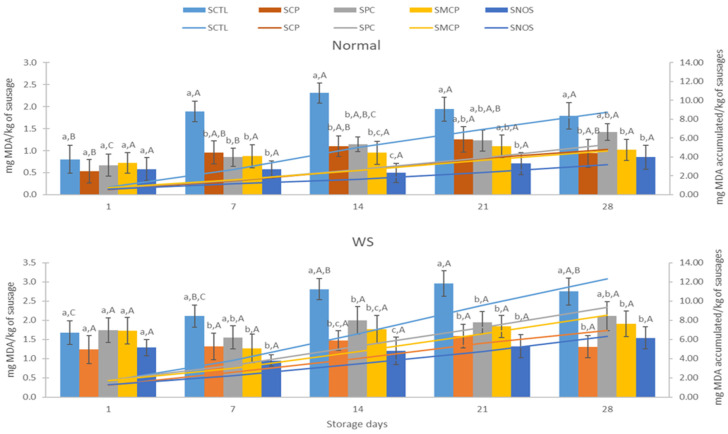
Effect of the incorporation of cactus fruit mixtures (cactus berry and red prickly pear) on lipid oxidation (TBARS, mg MAD/kg meat) of sausages (cooked meat batters) made with normal or White Striping (WS) broiler breast meat, n = 9. Bars represent MDA production at each sampling day, and continuous lines denote accumulated MDA. SCTL: sausages made from Normal or WS broiler breast meat, without adding mixtures of cactus fruits (negative control). SCP: sausages made from Normal or WS broiler breast meat, with the addition of CP; SPC: sausages made from Normal or WS broiler breast meat, with the addition of PC. SMCP: sausages made from Normal or WS broiler breast meat, with the addition of MPC. SNOS: sausages made from Normal or WS broiler breast meat, with the addition of 120 mg of nitrites/kg of batter. ^a,b,c^: Different letters between treatments in each day and in the same type of broiler breast meat indicate differences (*p* < 0.05). ^A,B,C^: Different superscripts between days in the same treatment in each type of broiler breast meat indicate differences (*p* < 0.05).

The oxidative effects in the emulsions were significant for both Normal and WS meat (*p* < 0.05). Lipid oxidation in batter emulsions (Figure 2) and sausages (Figure 3) varied significantly among treatments (*p* < 0.05). Regarding BCTL, it presented the highest oxidation in both types of meat (~2.5 mg MDA/kg for normal and ~3.5 mg MDA/kg for WS). BNOS and BCP were among the lowest in Normal and WS, respectively. Processing steps such as cutting, deboning, grinding, or cooking promote oxidation by disrupting muscle membranes and releasing phospholipids, which subsequently interact with pro-oxidant factors such as oxygen, enzymes, or metals [10,34]. In addition, extrinsic factors such as temperature and light can accelerate oxidative reactions. High oxidation levels have also been observed in raw ground beef when not processed under vacuum [34]. This suggests that the lack of vacuum processing contributed to the elevated TBARS values observed in some of the treatments.

Overall, emulsions from WS meat with cactus mixtures presented higher oxidation than those from Normal meat. Regarding the cactus mixtures, BCP exhibited the lowest oxidation values (*p* < 0.05) in the emulsified batters for both types of meat. In contrast, BPC and BMCP showed similar values between both treatments within each meat type, resulting in oxidation levels slightly higher than BCP (*p* < 0.05). In general, batter emulsions from Normal meat showed that BPC and BMCP mixtures reached, on average, ~2.0 mg MDA/kg, while the lowest values were recorded in BCP and BNOS (≈1.2 mg MDA/kg, *p* < 0.05). This same behaviour, but with a different magnitude, was observed for WS; the lowest values were observed in BCP and BNOS (~2.4 mg MDA/kg on average).

These findings align with the in vitro AA results (DPPH^•^, ABTS^•+^, FRAP) of meat emulsions (Table 5), where Normal and WS emulsions, particularly BCP, displayed stronger antioxidant activity in DPPH^•^ and FRAP compared to the other mixtures (BPC and BMCP). Given the high proportion of cactus berry in this mixture (75% cactus berry + 25% red prickly pear), it is rich in flavonoids, which are known to inactivate alkyl peroxyl radicals and superoxides, thereby preventing lipid oxidation [42,44]. The redox properties of cactus berry and prickly pear polyphenols play a crucial role in neutralising free radicals, reducing singlet oxygen, and decomposing peroxides [69,70]. In particular, compounds such as betanin and indicaxanthin act through hydroxyl groups that donate hydrogen atoms or electrons to free radicals and reactive oxygen species [15,23].

In general, sausages made with normal meat, TBARS values from SCTL increased (*p* < 0.05) and showed greater oxidation over the storage period (Figure 3). In contrast, SNOS effectively controlled the oxidative process, maintaining low levels throughout storage (*p* < 0.05).

Moreover, all TBARS values for each treatment increased between the first and seventh day of storage, with the three cactus mixtures and SNOS maintaining low oxidation on day 7. However, by day 14, SCP and SPC showed higher oxidation than SNOS (*p* < 0.05). Moreover, after 21 days of storage, the concentrations of TBARS in SCP, SPC, and SMCP were similar (*p* > 0.05) between the three cactus mixtures. At the end of the storage, SCP, SMCP, and SNOS showed values of ≈1.2 mg MDA/kg, indicating that both mixtures (SCP and SMCP) were effective in controlling oxidation at the same level as nitrites. On the other hand, in WS sausages with mixtures, it can be observed that all treatments remained stable until day 7 of storage. Moreover, all values increased between days 7 and 14 of storage, probably as a result of lipid oxidation [34], with SNOS showing the lowest TBARS values on day 14. From day 14 onwards, the treatments with the mixtures remain almost constant. However, at the end of the storage period, lipid oxidation was lowest (*p* < 0.05) in SCP, SMCP, and SNOS. In the case of SCTL, the TBARS value was higher at the end of storage compared with the other treatments (*p* < 0.05). Therefore, at the end of the storage period, the Normal and WS meat with the incorporation of the cactus mixtures showed MDA values below 2.0 mg of MDA/kg meat. In this sense, TBARS values > 2.0 are usually associated with detectable oxidised odour and flavour of cooked samples [34].

## 4. Conclusions

The study demonstrated that powders from cactus berry (*Myrtillocactus geometrizans*) and red prickly pear (*Opuntia ficus-indica* L. Mill.) are effective natural colourants and antioxidants in chicken sausages, particularly those formulated with White Striping (WS) meat. Regarding the characterisation of extracts, red prickly pear showed greater antioxidant activity than cactus berry, whereas cactus berry imparted a more intense red colour. Mixtures of both fruits enhanced antioxidant capacity and colour stability, effectively inhibiting lipid oxidation and maintaining redness for up to 28 days under refrigerated, vacuum storage. Cooking further increased antioxidant activity, even surpassing the effect of nitrites. Treatments with 75% cactus berry and 25% red prickly pear (CP) showed the most pronounced benefits. Overall, these findings support the use of cactus-derived fruit powders as viable natural alternatives to synthetic nitrites, offering improved oxidative stability and colour retention. Future research should address sensory acceptance and the preservation of bioactive compounds in industrial-scale applications.

## Figures and Tables

**Table 1:** Description of experimental treatments and analyses carried out.

	Cactus Fruits and Mixtures ^A^		Meat Products	
		Broiler Breast Meat ^B^	Batters ^C^	Sausages ^D^
Experimental treatments	Powders: C and P Mixtures: CP (75% C + 25% C), PC (75% P + 25% C), MCP (50% C + 50% P)	Normal and WS meat	BCP, BPC, BMCP, BCTL, and BNOS	SCP, SPC, SMCP, SCTL, and SNOS
Analysis carried out (sampling days)	CPT (0) CFT (0) Antioxidant activity (0) Instrumental colour (0)	Chemical composition ^E^ (0) pH (0) Instrumental colour (0) Antioxidant activity (0) Texture (0) TBARS (0)	Antioxidant activity (0) Instrumental colour (0) TBARS (0)	pH (1, 7, 14, 21, 28) Antioxidant activity (1, 7, 14, 21, 28) Instrumental colour (1, 7, 14, 21, 28) TBARS (1, 7, 14, 21, 28)

^A^ Evaluation in vitro considering two solvents (distilled water and ethanol at 75%), and extracts were prepared at 0.2 g/mL. ^B^ Raw meat evaluation, both (Normal and WS) meats were evaluated separately. ^C^ Batters (raw meat emulsions) were made with one of two broiler breast meats in order to apply one of five treatments. Powder mixtures were added at 5% (*w*/*w*) in formulations. Prefix B-in names’ treatments were used. ^D^ Previous batters were cooked to reach an internal temperature of 72 °C. Then, sausages (cooked batters) were refrigerated at 1.5 °C for 28 days. Prefix S-in names’ treatments were used. ^E^ Chemical composition included moisture, crude protein, fat and ash. Antioxidant activity was evaluated using DPPH^•^, ABTS^•+^ and FRAP methods. Instrumental colour was evaluated (L*, a*, b*, Chroma and Hue values).

**Table 2:** Total polyphenol content (TPC), total flavonoid content (TFC), antioxidant activity (DPPH•, ABTS•+, FRAP), and colour (L*, a*, b*) of cactus berry (C) and red prickly pear (P) dry products in two solvents (ethanolic and aqueous).

	Treatments			
	C		P	
	Ethanolic	Aqueous	Ethanolic	Aqueous
TPC ^X^	0.2 ± 0.03 ^c^	1.4 ± 0.04 ^b^	0.2 ± 0.03 ^c^	1.7 ± 0.05 ^a^
TFC ^Y^	0.1 ± 0.04 ^b^	0.6 ± 0.14 ^a^	0.1 ± 0.05 ^b^	0.6 ± 0.09 ^a^
DPPH^● Z^	29.6 ± 0.28 ^c^	49.9 ± 0.24 ^b^	27.9 ± 0.12 ^d^	80.4 ± 0.40 ^a^
ABTS^●+ Z^	351.3 ± 13.26 ^c^	796.8 ± 13.74 ^b^	411.7 ± 3.89 ^c^	1020.9 ± 8.06 ^a^
FRAP ^Z^	312.5 ± 6.15 ^a^	100.7 ± 3.65 ^d^	191.2 ± 2.04 ^c^	225.9 ± 3.78 ^b^
L*	80.4 ± 1.82 ^a^	56.1 ± 1.11 ^c^	80.5 ± 1.12 ^a^	62.3 ± 0.88 ^b^
a*	−5.6 ± 0.11 ^c^	41.2 ± 1.14 ^a^	−5.2 ± 0.03 ^c^	14.8 ± 0.21 ^b^
b*	10.8 ± 0.19 ^b^	−1.4 ± 0.09 ^c^	10.3 ± 0.14 ^b^	29.1 ± 0.23 ^a^

All values are mean ± standard deviation, n = 9. ^a,b,c,d^: Different superscripts in the same row indicate differences (*p* < 0.05). ^X^: Milligrams of gallic acid equivalents (mg GAE/100 g). ^Y^: Milligrams of quercetin equivalents (mg QE/100 g). ^Z^: μmol Trolox equivalent/g.

**Table 3:** Total polyphenol content (TPC), total flavonoid content (TFC), antioxidant activity (DPPH^•^, ABTS^•+^, FRAP), and colour (L*, a*, b*) of mixtures of cactus berry (C) and red prickly pear (P) in two solvents (ethanolic and aqueous), n = 9.

BBM	Treatments					
	CP		PC		MCP	
	Ethanolic	Aqueous	Ethanolic	Aqueous	Ethanolic	Aqueous
TPC ^X^	0.6 ± 0.16 ^d^	1.3 ± 0.19 ^c^	0.5 ± 0.10 ^e^	1.5 ± 0.15 ^a^	0.6 ± 0.11 ^d^	1.4 ± 0.13 ^b^
TFC ^Y^	0.3 ± 0.15 ^b^	1.2 ± 0.12 ^a^	0.2 ± 0.14 ^b^	0.4 ± 0.18 ^b^	0.1 ± 0.13 ^b^	0.5 ± 0.16 ^b^
DPPH^● Z^	28.1 ± 0.16 ^d^	59.3 ± 0.32 ^c^	25.2 ± 0.28 ^e^	77.6 ± 0.29 ^a^	24.54 ± 0.36 ^e^	61.0 ± 0.36 ^b^
ABTS^●+ Z^	167.7 ± 14.61 ^f^	926.9 ± 22.22 ^b^	384.2 ± 16.26 ^d^	985.2 ± 12.35 ^a^	251.63 ± 11.48 ^e^	465.7 ± 15.90 ^c^
FRAP ^Z^	303.7 ± 2.26 ^b^	156.0 ± 7.18 ^e^	190.0 ± 2.65 ^d^	221.5 ± 4.42 ^c^	336.57 ± 1.97 ^a^	128.3 ± 2.34 ^f^
L*	81.9 ± 0.22 ^a^	55.4 ± 0.16 ^c^	81.4 ± 0.42 ^a^	60.7 ± 0.63 ^b^	82.06 ± 0.29 ^a^	61.5 ± 0.34 ^b^
a*	−5.9 ± 0.25 ^d^	37.4 ± 0.31 ^a^	−5.1 ± 0.34 ^d^	23.3 ± 0.79 ^c^	−5.74 ± 0.10 ^d^	27.1 ± 0.62 ^b^
b*	11.4 ± 0.17 ^b^	5.2 ± 0.08 ^d^	9.4 ± 0.14 ^c^	20.1 ± 0.53 ^a^	11.08 ± 0.28 ^b^	10.0 ± 0.22 ^c^

CP: 75% of Cactus berry + 25% of Red prickly pear. PC: 75% of Red prickly pear + 25% of Cactus Berry; MCP: 50% of Cactus Berry + 50% of Red prickly pear. All values are mean ± standard deviation. ^a,b,c,d,e,f^: Different superscripts in the same row indicate differences (*p* < 0.05). ^X^: Milligrams of gallic acid equivalents (mg GAE/100 g). ^Y^: Milligrams of quercetin equivalents (mg QE/100 g). ^Z^: μmol Trolox equivalent/g.

**Table 4:** Chemical composition (%), pH, instrumental colour (L*, a*, b*), shear force (kg/cm^2^), and antioxidant activity (DPPH^●^, ABTS^•+^, FRAP) in broiler breast meat.

		Type of Broiler Breast Meat	
		Normal	WS
Chemical composition	Moisture	72.9 ± 0.22 ^a^	73.2 ± 0.17 ^a^
	Protein crude	19.0 ± 0.64 ^a^	18.5 ± 0.24 ^a^
	Fat	5.7 ± 0.04 ^b^	6.1 ± 0.04 ^a^
	Ash	1.3 ± 0.09 ^a^	1.3 ± 0.03 ^a^
pH		5.7 ± 0.06 ^b^	6.0 ± 0.03 ^a^
Instrumental colour	L*	64.8 ± 1.21 ^a^	62.9 ± 0.81 ^a^
	a*	5.2 ± 0.22 ^b^	6.8 ± 0.19 ^a^
	b*	26.6 ± 0.48 ^a^	17.6 ± 0.70 ^b^
	Chroma	27.1 ± 0.42 ^a^	19.0 ± 0.64 ^b^
	Hue angle	78.8 ± 0.67 ^a^	69.2 ± 1.00 ^b^
Texture	Shear force	1.8 ± 0.15 ^a^	1.3 ± 0.07 ^b^
Antioxidant activity	DPPH^● Y^	30.0 ± 5.33 ^b^	62.3 ± 1.90 ^a^
	ABTS^●+ Y^	469.0 ± 4.25 ^b^	751.2 ± 6.91 ^a^
	FRAP ^Y^	52.3 ± 1.94 ^b^	64.6 ± 1.90 ^a^
Lipid oxidation	TBARS ^Z^	0.6 ± 0.09 ^b^	0.9 ± 0.06 ^a^

All values are mean ± standard deviation, n = 3. ^a,b^: Different superscripts in the same row indicate differences (*p* < 0.05). WS: Breast chicken with a Severe grade of White Striping myopathy. ^Y^: μmol Trolox equivalent/g. ^Z^: mg MDA/kg.

**Table 5:** Effect of the incorporation of mixtures of cactus fruits on the antioxidant activity (DPPH^•^, ABTS^•+^, FRAP) and instrumental colour (L*, a*, b*) in the batters (B) from normal and White Striping (WS) broiler breast meat (BBM).

BBM		Treatments				
		BCTL	BCP	BPC	BMCP	BNOS
Normal	DPPH^● Z^	31.6 ± 1.66 ^c^	65.2 ± 1.55 ^a^	53.0 ± 3.60 ^b^	53.6 ± 3.00 ^b^	52.4 ± 7.70 ^b^
	ABTS^●+ Z^	574.2 ± 9.02 ^d^	513.0 ± 12.91 ^d^	706.8 ± 17.55 ^b^	989.4 ± 13.48 ^a^	649.2 ± 27.27 ^c^
	FRAP ^Z^	204.1 ± 0.57 ^a^	181.6 ± 4.67 ^b^	150.3 ± 1.86 ^d^	172.1 ± 3.97 ^c^	207.4 ± 2.65 ^a^
	L*	90.2 ± 0.05 ^b^	81.3 ± 0.06 ^d^	83.0 ± 0.22 ^c^	83.1 ± 0.53 ^c^	92.7 ± 0.19 ^a^
	a*	5.1 ± 0.06 ^c^	11.7 ± 0.12 ^a^	10.6 ± 0.13 ^b^	12.0 ± 0.25 ^a^	5.1 ± 0.30 ^c^
	b*	19.8 ± 0.13 ^a^	10.7 ± 0.06 ^d^	13.6 ± 0.10 ^b^	12.1 ± 0.20 ^c^	19.9 ± 0.02 ^a^
	Hue angle	75.6 ± 0.09 ^a^	42.5 ± 0.21 ^d^	52.1 ± 0.35 ^b^	45.2 ± 0.41 ^c^	75.7 ± 0.06 ^a^
WS	DPPH^● Z^	36.6 ± 6.03 ^c^	79.8 ± 5.60 ^a^	60.3 ± 6.43 ^b^	66.1 ± 2.04 ^b^	33.8 ± 1.45 ^c^
	ABTS^●+ Z^	643.4 ± 14.37 ^d^	1080.0 ± 6.42 ^b^	1180.4 ± 24.28 ^a^	1072.2 ± 14.32 ^b^	740.3 ± 24.04 ^c^
	FRAP ^Z^	140.6 ± 3.24 ^d^	201.8 ± 3.46 ^b^	162.3 ± 0.97 ^c^	161.09 ± 2.27 ^c^	232.0 ± 8.43 ^a^
	L*	95.4 ± 0.18 ^a^	84.7 ± 0.84 ^b^	83.4 ± 0.95 ^b^	84.4 ± 0.90 ^b^	95.0 ± 0.38 ^a^
	a*	2.9 ± 0.49 ^c^	11.3 ± 0.13 ^a^	11.2 ± 0.20 ^a^	11.5 ± 0.33 ^a^	4.4 ± 0.32 ^b^
	b*	20.1 ± 0.10 ^a^	12.9 ±0.76 ^b^	12.3 ± 0.28 ^b^	12.6 ± 0.65 ^b^	19.9 ± 0.19 ^a^
	Hue angle	81.7 ± 1.39 ^a^	48.3 ± 1.35 ^b^	47.6 ± 0.98 ^b^	47.2 ± 2.19 ^b^	77.7 ± 0.93 ^a^

All values are mean ± standard deviation, n = 9. ^a,b,c,d^: Different superscripts in the same row indicate differences (*p* < 0.05). WS: Breast chicken with a severe grade of white striping myopathy. BCTL: batter made from broiler breast meat, without adding mixtures of cactus fruits (negative control). BCP: batter made from broiler breast meat, with the addition of CP. BPC: batter made from broiler breast meat, with the addition of PC. BMCP: batter made from broiler breast meat with the addition of MPC. BNOS: batter made from breast meat chicken with the addition of 120 mg of nitrites/kg of batter. ^Z^: μmol Trolox equivalent/g.

**Table 6:** Effect of the incorporation of cactus fruits mixtures (CP, PC and MCP) on the pH and instrumental colour (L*, a*, b*) changes in the sausages (cooked emulsion batters) from Normal broiler breast meat.

		Treatments				
	Day	SCTL	SCP	SPC	SMCP	SNOS
pH	1	6.5 ± 0.03 ^a,C^	6.4 ± 0.08 ^a,b,C^	6.3 ± 0.05 ^b,B^	6.3 ± 0.06 ^b,B,C^	6.5 ± 0.03 ^a,A,B^
	7	6.4 ± 0.04 ^a,C,D^	6.1 ± 0.07 ^b,D^	5.9 ± 0.11 ^c,C^	6.2 ± 0.07 ^b,C^	6.4 ± 0.07 ^a,B^
	14	6.3 ± 0.05 ^a,b,D^	6.4 ± 0.06 ^a,C^	6.2 ± 0.05 ^b,c,B^	6.3 ± 0.01 ^a,b,B,C^	6.1 ± 0.14 ^c,C^
	21	6.8 ± 0.06 ^a,B^	6.6± 0.09 ^b,B^	6.3 ± 0.09 ^c,B^	6.4 ± 0.09 ^c,B,C^	6.3 ± 0.09 ^c,B,C^
	28	7.1 ± 0.04 ^a,A^	6.8 ± 0.11 ^b,A^	6.7 ± 0.12 ^b,A^	6.6 ± 0.10 ^b,A^	6.7 ± 0.12 ^b,A^
L*	1	96.0 ± 0.21 ^a,A^	84.6 ± 0.41 ^c,A,B^	86.4 ± 0.34 ^b,B^	85.1 ± 0.40 ^b,c,B^	96.1 ± 0.25 ^a,A^
	7	94.9 ± 0.18 ^a,B^	85.2 ± 0.46 ^c,A,B^	87.2 ± 0.47 ^b,A^	85.9 ± 0.49 ^c,A,B^	94.5 ± 0.25 ^a,C^
	14	95.5 ± 0.22 ^a,A^	85.7 ± 0.48 ^c,A^	87.2 ± 0.38 ^b,A^	85.8 ± 0.47 ^c,A,B^	95.3 ± 0.30 ^a,B^
	21	94.4 ± 0.31 ^a,B,C^	84.1 ± 0.56 ^c,B,C^	86.8 ± 0.36 ^b,A,B^	86.2 ± 0.38 ^b,A^	94.7 ± 0.27 ^a,B,C^
	28	94.1 ± 0.24 ^b,C^	83.9 ± 0.67 ^d,C^	86.1 ± 0.41 ^c,B^	85.3 ± 0.43 ^c,A,B^	95.8 ± 0.25 ^a,A,B^
a*	1	3.8 ± 0.21 ^c,A^	11.0 ± 0.19 ^a,A^	10.2 ± 0.48 ^b,A^	10.7 ± 0.30 ^a,b,A^	3.6 ± 0.82 ^c,A^
	7	2.3 ± 0.28 ^c,B^	10.1 ± 0.19 ^a,B^	9.1 ± 0.12 ^b,B^	9.7 ± 0.31 ^a,b,B^	2.5 ± 0.47 ^c,A^
	14	3.4 ± 0.57 ^c,A^	9.5 ± 0.14 ^a,C^	8.4 ± 0.13 ^b,C^	9.0 ± 0.35 ^a,b,B,C^	3.2 ± 0.71 ^c,A^
	21	3.1 ± 0.37 ^b,A,B^	8.8 ± 0.21 ^a,D^	8.4 ± 0.23 ^a,C^	8.2 ± 0.41 ^a,C^	3.1 ± 0.65 ^b,A^
	28	3.5 ± 0.53 ^c,A^	8.2 ± 0.24 ^a,E^	6.8 ± 0.21 ^b,D^	7.5 ± 0.39 ^a,b,C^	2.9 ± 0.52 ^c,A^
b*	1	18.9 ± 0.31 ^a,A^	11.6 ± 0.29 ^d,C^	13.7 ± 0.29 ^b,B^	12.4 ± 0.58 ^c,C^	18.9 ± 0.25 ^a,A^
	7	12.6 ± 0.25 ^c,B^	18.4 ± 0.32 ^a,C^	14.3 ± 0.31 ^b,A,B^	18.2 ± 0.47 ^a,B^	12.0 ± 0.31 ^c,B^
	14	12.9 ± 0.35 ^c,B^	18.7 ± 0.27 ^a,C^	14.4 ± 0.27 ^b,A^	18.1 ± 0.50 ^a,B^	12.4 ± 0.24 ^c,B^
	21	13.2 ± 0.41 ^d,B^	20.6 ± 0.31 ^a,B^	14.6 ± 0.34 ^c,A^	19.5 ± 0.48 ^b,A^	9.5 ± 0.23 ^e,C^
	28	12.7 ± 0.21 ^d,B^	21.4 ± 0.33 ^a,A^	14.8 ± 0.31 ^c,A^	20.1 ± 0.52 ^b,A^	8.3 ± 0.26 ^e,D^
Hue angle	1	78.6 ± 3.10 ^a,A^	46.5 ± 1.09 ^c,C^	53.3 ± 0.48 ^b,D^	49.2 ± 2.34 ^b,c,C^	79.2 ± 2.24 ^a,A^
	7	79.7 ± 2.18 ^a,A^	61.2 ± 1.23 ^b,B^	57.5 ± 0.32 ^c,C^	61.9 ± 2.25 ^b,B^	78.2 ± 1.42 ^a,A^
	14	75.2 ± 1.63 ^a,A^	63.1 ± 1.00 ^b,B^	59.7 ± 0.24 ^c,B^	63.6 ± 2.02 ^b,B^	75.5 ± 2.42 ^a,A,B^
	21	76.8 ± 2.30 ^a,A^	66.9 ± 2.30 ^c,A^	60.1 ± 0.31 ^d,B^	67.2 ± 1.98 ^c,A,B^	71.9 ± 2.32 ^b,B^
	28	74.6 ± 2.65 ^a,A^	69.0 ± 2.45 ^b,A^	65.3 ± 0.29 ^b,A^	69.5 ± 2.76 ^b,A^	69.6 ± 2.54 ^b,B^

All values are mean ± standard deviation, n = 9. ^a,b,c,d,e^: Different superscripts in the same row indicate differences (*p* < 0.05). ^A,B,C,D,E^: Different superscripts in between days on the same variable and treatment indicate differences (*p* < 0.05). SCTL: sausages made from normal broiler breast meat, without adding mixtures of cactus fruits (negative control). SCP: sausages made from normal broiler breast meat, with the addition of CP. SPC: sausages made from normal broiler breast meat, with the addition of PC. BMCP: sausages made from normal broiler breast meat, with the addition of MPC. SNOS: sausages made from normal broiler breast meat, with the addition of 120 mg of nitrites/kg of batter.

**Table 7:** Effect of the incorporation of cactus fruit mixtures (CP, PC, and MCP) on the pH and instrumental colour (L*, a*, b*) changes in the sausages (cooked batters) from White Striping (WS) broiler breast meat during refrigerated display.

		Treatments				
	Day	SCTL	SCP	SPC	SMCP	SNOS
pH	1	6.6 ± 0.16 ^a,B,C^	6.3 ± 0.02 ^b,B^	6.4 ± 0.05 ^b,A^	6.3 ± 0.04 ^b,A,B^	6.4 ± 0.02 ^b,A^
	7	6.4 ± 0.21 ^a,C^	6.3 ± 0.08 ^a,B^	6.3 ± 0.16 ^a,A,B^	6.3 ± 0.09 ^a,A,B^	6.4 ± 0.08 ^a,A^
	14	5.9 ± 0.09 ^b,D^	6.3 ± 0.09 ^a,B^	6.1 ± 0.08 ^a,b,B^	5.9 ± 0.13 ^b,C^	6.2 ± 0.11 ^a,A^
	21	6.8 ± 0.10 ^a,B^	6.5 ± 0.14 ^b,A^	6.3 ± 0.10 ^b,c,A,B^	6.2 ± 0.09 ^c,B^	6.3 ± 0.09 ^b,c,A^
	28	7.3 ± 0.12 ^a,A^	6.6 ± 0.06 ^b,A^	6.5 ± 0.11 ^b,A^	6.5 ± 0.11 ^b,A^	6.4 ± 0.14 ^b,A^
L*	1	93.7 ± 0.60 ^a,A^	83.1 ± 0.30 ^c,A,B^	85.1 ± 0.33 ^b,B^	83.1 ± 0.46 ^c,C^	93.5 ± 0.40 ^a,B^
	7	93.0 ± 0.50 ^a,A^	83.4 ± 0.14 ^d,A^	86.2 ± 0.26 ^b,A^	84.1 ± 0.27 ^c,B^	93.5 ± 0.15 ^a,B^
	14	92.8 ± 0.53 ^b,A^	82.6 ± 0.46 ^e,B^	86.3 ± 0.43 ^c,A^	84.5 ± 0.31 ^d,A,B^	94.1 ± 0.15 ^a,A^
	21	92.6 ± 0.76 ^b,A^	82.3 ± 0.35 ^e,B^	86.5 ± 0.32 ^c,A^	84.8 ± 0.29 ^d,A,B^	94.3 ± 0.32 ^a,A^
	28	92.4 ± 0.87 ^b,A^	82.0 ± 0.29 ^e,B^	86.8 ± 0.35 ^c,A^	85.0 ± 0.35 ^d,A^	93.9 ± 0.28 ^a,A,B^
a*	1	3.2 ± 0.17 ^d,A^	10.4 ± 0.04 ^a,A^	9.3 ± 0.23 ^b,A^	10.2 ± 0.18 ^a,A^	4.0 ± 0.32 ^c,B^
	7	3.4 ± 0.06 ^d,A^	9.9 ± 0.15 ^a,B^	8.7 ± 0.16 ^b,B^	9.2 ± 0.08 ^a,C^	4.8 ± 0.18 ^c,A^
	14	3.3 ± 0.10 ^e,A^	9.0 ± 0.06 ^b,C^	8.1 ± 0.15 ^c,C^	9.9 ± 0.16 ^a,A^	4.9 ± 0.28 ^d,A^
	21	3.5 ± 0.16 ^e,A^	9.1 ± 0.21 ^b,C^	8.1 ± 0.26 ^c,C^	9.7 ± 0.26 ^a,B^	4.8 ± 0.26 ^d,A^
	28	3.4 ± 0.21 ^e,A^	8.9 ± 0.24 ^b,C^	7.9 ± 0.19 ^c,C^	9.7 ± 0.33 ^a,B^	4.6 ± 0.33 ^d,A,B^
b*	1	18.5 ± 0.21 ^a,B^	11.6 ± 0.15 ^d,B^	14.3 ± 0.11 ^b,B^	12.1 ± 0.44 ^c,C^	18.1 ± 0.08 ^a,B^
	7	18.2 ± 0.14 ^a,B^	12.3 ± 0.26 ^d,A^	14.3 ± 0.54 ^c,B^	15.6 ± 1.79 ^b,A^	17.7 ± 0.05 ^a,C^
	14	19.1 ± 0.43 ^a,A,B^	11.8 ± 0.25 ^e,A^	15.0 ± 0.37 ^c,B^	13.2 ± 0.27 ^d,B,C^	18.0 ± 0.19 ^b,B,C^
	21	18.8 ± 0.32 ^a,A,B^	11.9 ± 0.31 ^e,A^	15.3 ± 0.27 ^c,A,B^	13.9 ± 0.65 ^d,B^	17.8 ± 0.23 ^b,B^
	28	19.2 ± 0.28 ^a,A^	11.2 ± 0.29 ^d,B^	15.9 ± 0.29 ^b,A^	14.4 ± 0.52 ^c,A,B^	18.5 ± 0.29 ^a,A^
Hue angle	1	80.2 ± 0.61 ^a,A^	46.4 ± 0.28 ^e,C^	56.9 ± 0.82 ^c,C^	49.8 ± 1.52 ^d,B^	77.5 ± 0.93 ^b,A^
	7	79.4 ± 0.18 ^a,A^	51.2 ± 0.53 ^d,B^	58.7 ± 0.93 ^c,C^	59.5 ± 2.40 ^c,A^	74.8 ± 0.19 ^b,B^
	14	80.2 ± 0.44 ^c,A^	52.7 ± 0.46 ^a,A^	61.6 ± 1.03 ^b,B^	53.1 ± 1.65 ^a,B^	74.8 ± 0.46 ^a,B^
	21	79.5 ± 0.36 ^a,A^	52.6 ± 0.21 ^e,A^	62.1 ± 0.66 ^c,A,B^	55.1 ± 2.21 ^d,A,B^	74.9 ± 0.65 ^b,B^
	28	80.0 ± 0.41 ^a,A^	51.5 ± 0.34 ^e,B^	63.6 ± 0.72 ^c,A^	56.0 ± 2.65 ^d,A,B^	76.0 ± 0.71 ^b,B^

All values are mean ± standard deviation, n = 9. ^a,b,c,d,e^: Different superscripts in the same row indicate differences (*p* < 0.05). ^A,B,C^: Different superscripts in between days on the same variable and treatment indicate differences (*p* < 0.05). SCTL: sausages made from WS broiler breast meat, without adding mixtures of cactus fruits (negative control); SCP: sausages made from WS broiler breast meat, with the addition of CP. SPC: sausages made from WS broiler breast meat, with the addition of PC. BMCP: sausages made from WS broiler breast meat, with the addition of MPC. SNOS: sausages made from WS broiler breast meat, with the addition of 120 mg of nitrites/kg of batter.

**Table 8:** Effect of the incorporation of cactus fruits mixtures (CP, PC, and MCP) on antioxidant activity changes of sausages (cooked batters) from Normal and White Striping (WS) broiler breast meat (BBM) during refrigerated display.

					Treatments		
BBM		Days	SCTL	SCP	SPC	SMCP	SNOS
Normal	DPPH^● Z^	1	40.7 ± 3.74 ^e,A^	85.5 ± 1.41 ^a,A^	60.1 ± 3.13 ^d,A^	67.7 ± 0.85 ^c,A^	77.9 ± 2.37 ^b,A^
		7	27.0 ± 1.25 ^d,B^	65.0 ± 2.20 ^a,B^	57.5 ± 1.81 ^b,A^	53.8 ± 1.47 ^b,B^	45.2 ± 2.07 ^c,B^
		14	23.7 ± 1.41 ^d,B,C^	35.9 ± 1.48 ^b,C^	30.9 ± 1.18 ^c,C^	51.8 ± 5.11 ^a,B,C^	30.8 ± 1.61 ^c,C^
		21	20.5 ± 2.36 ^c,C,D^	36.4 ± 1.67 ^b,C^	35.8 ± 1.42 ^b,B^	48.2 ± 1.92 ^a,C^	24.7 ± 1.78 ^c,D^
		28	18.6 ± 1.94 ^c,D^	31.5 ± 2.40 ^b,D^	33.4 ± 2.22 ^b,B,C^	46.4 ± 2.22 ^a,C^	19.6 ± 1.62 ^c,E^
	ABTS^●+ Z^	1	777.7 ±19.89 ^b,A^	894.5 ± 13.68 ^a,D^	882.1 ± 12.91 ^a,D^	881.3 ± 16.67 ^a,A^	787.6 ± 7.03 ^b,E^
		7	732.1 ± 22.75 ^c,A^	914.6 ± 14.18 ^a,D^	928.0 ± 14.64 ^a,C^	878.1 ± 18.77 ^b,A^	885.0 ± 14.90 ^b,D^
		14	610.9 ± 15.21 ^e,B^	992.7 ± 11.65 ^b,C^	1075.0 ± 19.36 ^a,B^	868.4 ± 22.00 ^d,A^	951.1 ± 16.25 ^c,C^
		21	542.6 ± 31.23 ^d,C^	1040.1 ± 20.13 ^b,B^	1183.6 ± 24.34 ^a,A^	864.3 ± 26.98 ^c,A^	1060.6 ± 26.51 ^b,B^
		28	474.4 ± 24.35 ^d,D^	1100.4 ± 25.43 ^b,A^	1210.3 ± 27.43 ^a,A^	856.5 ± 19.56 ^c,A^	1170.5 ± 18.48 ^a,A^
	FRAP ^Z^	1	217.2 ± 8.40 ^c,A^	273.9 ± 6.53 ^a,A^	192.7 ± 3.27 ^d,A^	192.4 ± 1.47 ^d,A^	262.3 ± 2.55 ^b,A^
		7	196.3 ± 3.66 ^a,B^	192.8 ± 2.67 ^a,B^	190.8 ± 4.31 ^a,A^	195.6 ± 2.48 ^a,A^	175.3 ± 5.22 ^b,B^
		14	117.7 ± 2.93 ^d,C^	180.6 ± 1.86 ^a,C^	127.2 ± 6.89 ^c,C^	170.8 ± 1.42 ^b,B^	122.7 ± 3.99 ^c,d,C^
		21	80.7 ± 5.43 ^e,D^	159.1 ± 7.42 ^b,D^	140.5 ± 5.43 ^c,B^	177.1 ± 3.43 ^a,B^	111.4 ± 6.43 ^d,D^
		28	62.4 ±6.22 ^e,E^	147.8 ± 4.35 ^b,E^	134.8 ± 4.21 ^c,B,C^	175.4 ± 5.38 ^a,B^	94.7 ± 5.32 ^d,E^
WS	DPPH^● Z^	1	43.7 ± 1.71 ^d,A^	88.6 ± 1.29 ^a,A^	62.5 ± 1.62 ^c,A^	69.6 ± 2.06 ^b,A^	67.6 ± 1.65 ^b,A^
		7	29.7 ± 1.32 ^d,B^	75.5 ± 2.33 ^a,B^	59.1 ± 1.82 ^c,A^	66.0 ± 1.15 ^b,A,B^	55.4 ± 1.25 ^c,B^
		14	25.0 ± 0.44 ^d,C^	68.8 ± 3.06 ^a,C^	54.3 ± 1.83 ^c,B^	64.7 ± 1.56 ^b,B^	24.9 ± 0.55 ^d,C^
		21	23.1 ± 1.78 ^d,C,D^	66.9 ± 1.87 ^a,C^	52.4 ± 2.27 ^c,B^	61.9 ± 1.86 ^b,B^	26.1 ± 2.32 ^d,C^
		28	20.6 ± 0.96 ^c,D^	64.6 ± 2.14 ^a,C^	51.5 ± 2.03 ^b,B^	62.3 ± 1.96 ^a,B^	23.0 ± 1.76 ^c,C^
	ABTS^●+ Z^	1	682.7 ± 25.15 ^c,C^	1014.9 ± 12.54 ^b,A^	1076.8 ± 32.60 ^a,A^	1004.7 ± 25.61 ^b,A^	990.6 ± 21.74 ^b,A^
		7	793.9 ± 19.90 ^b,B^	1010.1 ± 16.57 ^a,A^	1028.4 ± 24.81 ^a,A^	1056.7 ± 32.29 ^a,A^	936.6 ± 15.88 ^a,A^
		14	856.8 ± 12.53 ^c,A^	1004.0 ± 19.68 ^a,A^	919.8 ± 13.95 ^b,B^	1012.7 ± 31.65 ^a,A^	1026.2 ± 29.04 ^a,A^
		21	873.9 ± 23.11 ^b,A^	1003.9 ± 20.45 ^a,A^	855.4 ± 27.54 ^b,C^	1012.0 ± 22.45 ^a,A^	1021.5 ± 25.67 ^a,A^
		28	890.7 ± 24.34 ^b,A^	1002.8 ± 23.35 ^a,A^	874.1 ± 22.35 ^b,B,C^	1012.8 ± 19.56 ^a,A^	1025.8 ± 30.23 ^a,A^
	FRAP ^Z^	1	212.8 ± 2.88 ^d,A^	253.8 ± 5.33 ^b,A^	254.7 ± 4.03 ^b,A^	269.7 ± 1.42 ^a,A^	243.1 ± 1.53 ^c,A^
		7	178.3 ± 5.11 ^d,B^	231.3 ± 3.35 ^c,B^	242.4 ± 1.49 ^b,B^	262.5 ± 6.46 ^a,A,B^	233.3 ± 5.52 ^b,c,B^
		14	120.2 ± 1.68 ^c,C,D^	224.2 ± 4.74 ^b,B^	226.4 ± 1.77 ^b,C^	252.3 ± 4.78 ^a,B^	225.2 ± 2.93 ^b,B,C^
		21	123.1 ± 2.56 ^d,C^	218.3 ± 4.23 ^c,B,C^	228.1 ± 2.54 ^b,C^	255.5 ± 3.41 ^a,B^	224.2 ± 3.45 ^b,c,C^
		28	114.0 ± 3.11 ^d,D^	211.1 ± 4.76 ^c,C^	224.5 ± 2.96 ^b,C^	252.4 ± 3.69 ^a,B^	221.1 ± 4.14 ^b,C^

All values are mean ± standard deviation, n = 9. ^a,b,c,d,e^: Different superscripts in the same row indicate differences (*p* < 0.05). ^A,B,C,D,E^: Different superscripts in between days on the same variable and treatment indicate differences (*p* < 0.05). SCTL: sausages made from Normal or WS broiler breast meat, without adding mixtures of cactus fruits (negative control). SCP: sausages made from Normal or WS broiler breast meat, with the addition of CP; SPC: sausages made from Normal or WS broiler breast meat, with the addition of PC. SMCP: sausages made from Normal or WS broiler breast meat, with the addition of MPC. SNOS: sausages made from Normal or WS broiler breast meat, with the addition of 120 mg of nitrites/kg of batter. ^Z^: μmol Trolox equivalent/g.

## Data Availability

The original contributions presented in the study are included in the article. Further inquiries can be directed to the corresponding author.

## Abbreviations

The following abbreviations are used in this manuscript:

C	Cactus berry
P	Red prickly pear
CP	Mixture of 75% cactus berry and 25% Red prickly pear, treatment
PC	Mixture of 75% red prickly pear and 25% cactus berry, treatment
MCP	Mixture of 50% cactus berry and 50% Red prickly pear, treatment
CTL	Control, treatment
NOS	Nitrites, treatment
AA	Antioxidant activity
DPPH^•^	Radical DPPH
ABTS^•+^	Radical ABTS
FRAP	Ferric Reducing Ability of Plasma
TBARS	Thiobarbituric Reactive Substances
MDA	Malonaldehyde
TPC	Total Polyphenol Content
TFC	Total Flavonoid Content
GAE	Gallic acid Equivalents
QE	Quercetin Equivalents
BBM	Broiler breast meat
WS	White striping
B	Batter, as a prefix in treatments
S	Sausages, as a prefix in treatments
